# Role of Poly [ADP-ribose] Polymerase 1 in Activating the *Kirsten* *ras* (*KRAS*) Gene in Response to Oxidative Stress

**DOI:** 10.3390/ijms21176237

**Published:** 2020-08-28

**Authors:** Giorgio Cinque, Annalisa Ferino, Erik B. Pedersen, Luigi E. Xodo

**Affiliations:** 1 Department of Medicine, Laboratory of Biochemistry, P.le Kolbe 4, 33100 Udine, Italy; cinque.giorgio@spes.uniud.it (G.C.); annalisa.ferino@uniud.it (A.F.); 2 Nucleic Acid Center, Institute of Physics and Chemistry, University of Southern Denmark, DK-5230 Odense, Denmark; erik@sdu.dk

**Keywords:** G4 DNA, *KRAS*, oxidative stress, 8-oxoguanine, PARP-1, transcription factors, transcription regulation

## Abstract

In pancreatic Panc-1 cancer cells, an increase of oxidative stress enhances the level of 7,8-dihydro-8-oxoguanine (8OG) more in the *KRAS* promoter region containing G4 motifs than in non-G4 motif G-rich genomic regions. We found that H_2_O_2_ stimulates the recruitment to the *KRAS* promoter of poly [ADP-ribose] polymerase 1 (PARP-1), which efficiently binds to local G4 structures. Upon binding to G4 DNA, PARP-1 undergoes auto PARylation and thus becomes negatively charged. In our view this should favor the recruitment to the *KRAS* promoter of MAZ and hnRNP A1, as these two nuclear factors, because of their isoelectric points >7, are cationic in nature under physiological conditions. This is indeed supported by pulldown assays which showed that PARP-1, MAZ, and hnRNP A1 form a multiprotein complex with an oligonucleotide mimicking the *KRAS* G4 structure. Our data suggest that an increase of oxidative stress in Panc-1 cells activates a ROS-G4-PARP-1 axis that stimulates the transcription of *KRAS*. This mechanism is confirmed by the finding that when PARP-1 is silenced by siRNA or auto PARylation is inhibited by Veliparib, the expression of *KRAS* is downregulated. When Panc-1 cells are treated with H_2_O_2_ instead, a strong up-regulation of *KRAS* transcription is observed.

## 1. Introduction

Pancreatic ductal adenocarcinoma (PDAC) is one of the most lethal human cancers with a median survival of about 6–8 months after diagnosis [1]. This poor prognosis is ascribable to its high aggressive nature and its resistance to conventional chemotherapies. Recent studies have demonstrated that point mutations in the *KRAS* proto-oncogene are the initial step of the PDAC carcinogenesis [2]. In the mutated form, the oncogene reprograms the metabolism of PDAC by increasing the glycolytic flux, the glutamine and serine metabolism, in order to generate biomass and reducing power necessary for tumor growth [3,4,5]. The dependence of the metabolic pathways on specific oncogenes led to the concept of “oncogene addiction”, according to which cancer cells although depending on a number of genetic aberrations often develop a dependency on a particular oncogene [6,7]. As PDAC cells are addicted to mutant *KRAS*, this oncogene has become the focus of intense investigation [8]. In our laboratory we discovered that the *KRAS* promoter contains upstream of the transcription start site (TSS) a G-rich region adopting a G-quadruplex (or G4) structure recognized by several transcription factors [9,10,11]. The presence in the *KRAS* promoter of this unusual DNA conformation has been confirmed by ChIP-seq analysis carried out on transcriptionally active chromatin [12]. Although some hypotheses on the role of G4 DNA in gene regulation have been suggested, the data so far obtained are insufficient to propose a general mechanism for transcription regulation by G4 DNA [13,14]. In 2012 we discovered that PARP-1 binds to the *KRAS* G4 region located upstream of TSS [11]. While PARP-1 was originally described as a protein involved in DNA repair [15], several studies suggest that it has also a role in transcription regulation [16,17,18]. It has been reported that PARP-1 behaves as a modulator of chromatin, a regulator for DNA-binding transcription factors and DNA methylation [19]. Human PARP-1 is a 113 kDa protein composed by an amino-terminal DNA binding domain comprising two zinc-finger motifs, a B domain containing a nuclear localization signal and a caspase-3 cleavage site, a central auto-modification domain allowing protein–protein interactions and a C-terminal domain harboring a highly conserved sequence that forms the catalytic site [20,21]. PARP-1 binds and cleaves NAD^+^ to nicotinamide and ADP-ribose (ADPR), and it couples one or more ADPR units to acceptor proteins, including itself. These may become mono(ADP-ribosyl)ated or poly(ADP-ribosyl)ated: A modification called PARylation [20,21]. Most PARP-1 functions are localized in the nucleus, where the protein is involved in DNA repair, chromatin structure, transcription, translation and oxidative stress [15,19,21]. In the present work we focus on the role of PARP-1 in transcription regulation of *KRAS* in PDAC cells. We report that PARP-1 is associated to a G4-motif region of the *KRAS* promoter, in particular when the cellular levels of oxidative stress and 8-oxoguanine (8OG) are increased. We also found that PARP-1, upon binding to the *KRAS* promoter G4 structures, undergoes auto PARylation. This seems to induce the recruitment of the transcription factors hnRNP A1 and MAZ and the formation of the transcription pre-initiation complex. Previous studies from our and other laboratories showed that MAZ and hnRNP A1 bind to the promoter G4 region of *KRAS* and activate transcription [22,23,24,25,26,27]. In summary, in this study we have identified a ROS-G4-PARP-1 axis that controls the expression of *KRAS* under conditions of enhanced oxidative stress.

## 2. Results and Discussion

### 2.1. G4 Formation in the KRAS Promoter and Guanine Oxidation

**Figure 1:**
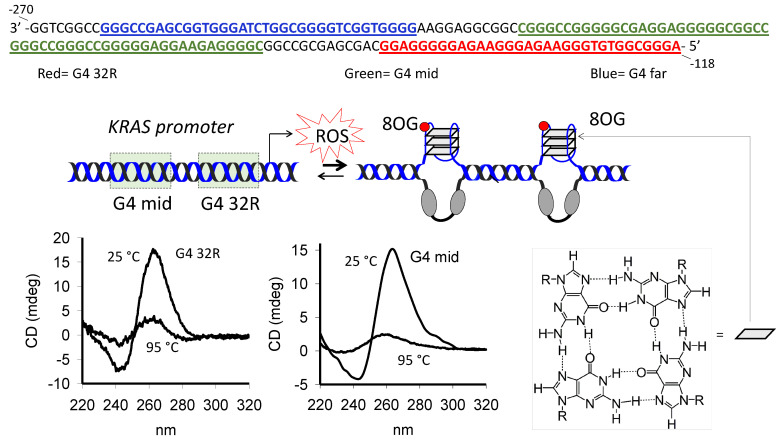
*KRAS* G4-motif sequences located upstream of the transcription start site (TSS). The G4 motifs are located in the non-coding strand between -118 and -270 from TSS: G4 32R (sequence in red, -118/-149); G4-mid (sequence in green, -163/-217; G4-far (sequence in blue, -229-264). G4 32R and G4 mid form G4 structures with a well-defined melting profile. The CD of these sequences (at 25 and 95 °C) suggest the formation of parallel G4 structures. The structure of a G-tetrad, where each guanine forms 4 hydrogen bonds with neighboring guanines, is shown.

**Figure 2:**
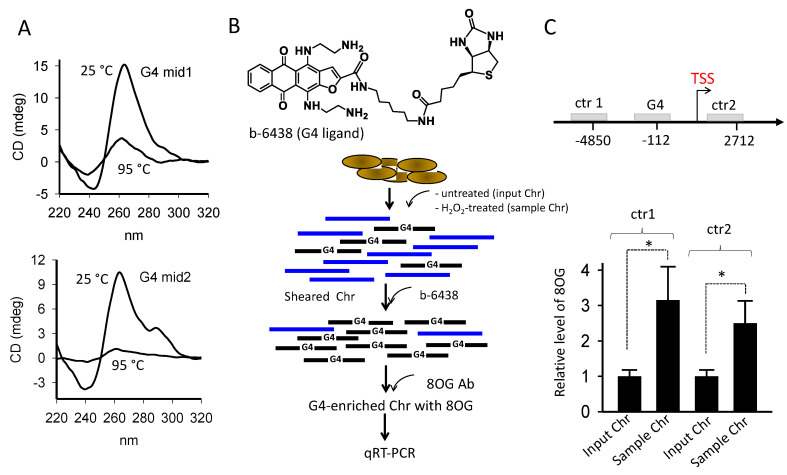
(**A**) CD spectra at 25 and 95 °C for the G4 mid1 and G4 mid2 sequences; (**B**) structure of biotinylated G4 ligand b-6438, which shows much more affinity for G4 DNA than duplex DNA, used in chromatin pull-down experiments. Pull down-ChIP experimental scheme. Chromatin fragments with G4 are depicted in black, fragments without G4 in blue; (**C**) the results of the pull down-ChIP experiment show that 8OG and the G4 motif co-localize in the region of the *KRAS* promoter containing G4 32R compared to control regions ctr1 and ctr2, located more than 4500 and 2500 bp away from TSS, respectively. Error bars from three experiments, Student *t*-test, (*) = *p* < 0.05.

The promoter of the human *KRAS* proto-oncogene contains, between −270 and −118 (3′→5′, non-coding strand) from the transcription start site (TSS), three G4 motifs hereafter called G4 32R (also called G4 near), G4 mid and G4 far (Figure 1) [9,28,29]. Previous studies have demonstrated that G4 32R and G4 mid form stable G4 structures under physiological conditions, whereas G4 far forms a G4 which is too weak for determining a thermal stability [28]. In Figure 1 we report typical CD spectra of G4 32R and G4 mid in 100 mM KCl, at 25 and 95 °C. Both exhibit a strong ellipticity at ~265 nm, in agreement with the formation of parallel G4 structures which have *T*_M_’s of 62 and 78 °C, respectively. DMS footprinting experiments showed that G4 32R forms a parallel tri-stacked G4 with a 1/1/12 topology, characterized by two 1-nt loops, a 12-nt loop and a T-bulge in one strand (Supplementary Information S3) [11]. A recent NMR study confirmed this structure, but showed that it is in equilibrium with a conformer containing a 11-nt loop, a folded-back guanine and 3-nt cap at the 3′-end [30]. By contrast, G4 mid comprising seven G-runs of at least three guanines can fold in different ways. In 100 mM KCl, G4 mid can fold into a quad-stacked G4 involving G-runs 1, 2, 5, and 6, as proposed in ref. [28]. It embeds, however, two contiguous G4 motifs, each of which is potentially able to form a tri-stacked G4. We called them G4 mid1 and G4 mid2 (Supplementary Information S4 and Table 1). The CD of G4 mid1 suggests that this sequence folds into a parallel G4. Instead, the CD of G4 mid2, showing a strong ellipticity at 260 nm and a shoulder at 295 nm, indicates that the sequence might adopt a mixed parallel/antiparallel G4. These G4 structures show *T*_M_’s in 100 mM KCl of 59 °C (G4 mid1) and 67 °C (G4 mid2) (Figure 2A, Supplementary Information S5). The presence in vivo of folded G4 structures in the *KRAS* promoter has been confirmed by a ChiP-seq analysis carried out on transcriptionally active chromatin [12].

It has been found that guanine, in particular in G-rich sequences, is susceptible to oxidation to 7,8-dihydro-8-oxoguanine (8OG), due to its low redox potential [31,32]. Indeed, the amount of 8OG in the genome of aerobic organisms is considered a biomarker for oxidative stress [33]. We previously have observed that pancreatic cancer cells (Panc-1, BxPC3, and MiaPaCa-2), due to their higher metabolic rate, show a higher basal level of 8OG than non-cancer HEK 293 cells [34]. We also discovered by ChIP experiments that when Panc-1 cells are treated with H_2_O_2_, 8OG is about 5-fold more abundant in the *KRAS* promoter region containing the G4-motifs than in G-rich non-G4 regions located at least 3000 bp away from TSS [35]. These data suggest that guanines in the G4-motifs are more exposed to oxidation than other guanines. Saito et al. [36] reported that contiguous guanines are characterized by a higher rate of oxidation than isolated guanines and that the 5′-G in a G-run shows the lowest ionization potential. To provide further support that 8OG is more abundant in G4 than non-G4 motifs, we performed a pull-down/ChIP experiment as outlined in Figure 2B. We synthesized a G4-bait, namely b-6438, by linking biotin to an anthrathiophenedione molecule binding to G4 much more than to duplex DNA [37].

We previously demonstrated that this molecule efficiently pulls down G4 strands from nucleic-acid mixtures [37]. Sheared chromatin obtained from untreated (input Chr) and H_2_O_2_-treated (sample Chr) Panc-1 cells were incubated with b-6438 and pulled down with streptavidin-coated magnetic beads. The recovered chromatin enriched with G4-containing fragments obtained from the input Chr and sample Chr were immuno-precipitated with an antibody specific for 8OG, and analyzed by qPCR with primers specific for the promoter G4 region and control ctr1 and ctr2 regions located in the *KRAS* gene (Figure 2B,C) [35]. The results showed that in sample Chr the *KRAS* promoter region harboring the G4 motifs contains, compared to ctr1 or ctr2, 3-fold more 8OG than in input Chr. These data further support the notion that ROS increase the level of 8OG more in the promoter region harboring the G4 motifs than in G-rich control regions unable to form G4. This is in keeping with an 8OG-Seq analysis showing that in the mouse genome, 8OG is 2-fold more abundant in gene promoters than in genic and intergenic regions [38,39]. In a previous work we asked if 8OG affects the topology and stability of the G4 formed by G4 32R [35]. In brief, we found that when the oxidative lesion lies in a loop (as in sequence **96**, Table 1), both the topology (1/1/12) and the stability (~62 °C) of the resulting G4 did not appreciably change. By contrast, when 8OG lies in the G-tetrad core (as in sequence **92**, Table 1), the damaged G-run is replaced by the additional G-run present in G4 32R, through an alternative folding. This is due to the fact that 8OG destabilizes G-tetrad [35] (Supplementary Information S6). The topology of G4 changed from 1/1/12 into 4/4/6 and the *T*_M_ decreases by ~10 °C [35]. These data show that the impact of 8OG on G4 depends on the position of the oxidized base within the G4 motif [40,41]. The effect of 8OG in G4 mid1 and G4 mid2 is similar to that observed with G4 32R. If we assume that G4 mid1 and G4 mid2 form, as observed for G4 32R, a G4 structure containing a 1-nt loop and a bulge in one strand, 8OG in G4 mid1^OX^ and G4 mid2^OX^ are expected to fall in the G4 core, destabilizing the structure. Indeed, we found that the *T*_M_’s of G4 mid1^OX^ and G4 mid2^OX^ are respectively 3 and 4 °C lower than the wild-type analogues (Table 1, Supplementary Information S5).

### 2.2. Under Oxidative Stress, PARP-1 Is Recruited to the KRAS Promoter where It Binds to G4 32R and G4 mid1 and G4 mid2 

**Figure 3:**
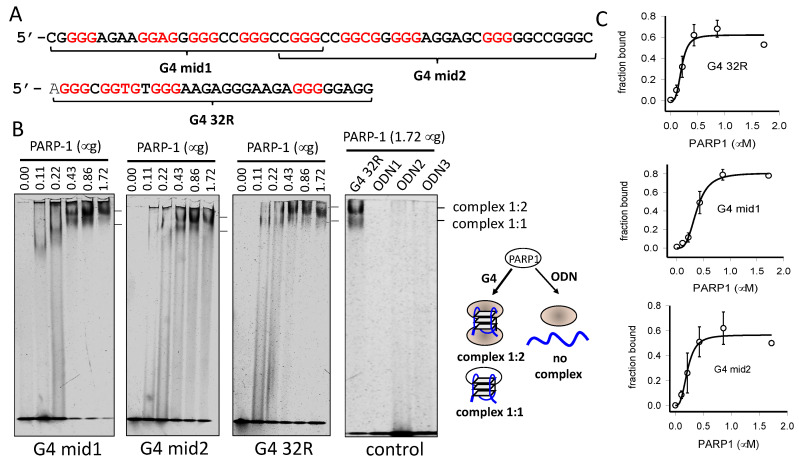

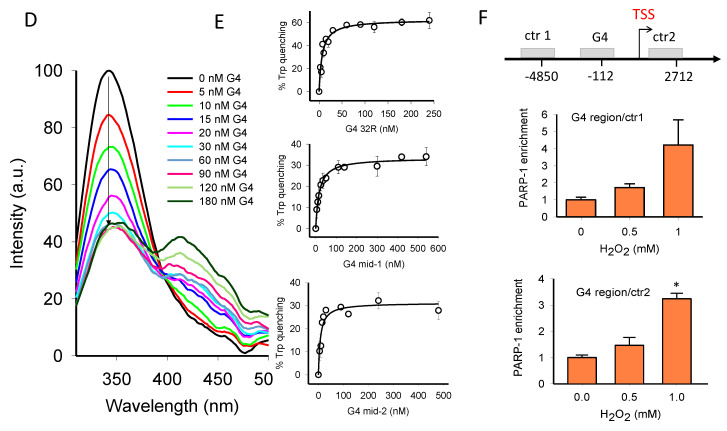
(**A**) Sequences of G4 32R, G4 mid1 and G4 mid2; (**B**) EMSA showing the binding of recombinant PARP-1 to Cy5.5-labelled G4-mid1, G4 mid2, G4 32R and control oligonucleotides ODN1, ODN2 and ODN3 (50 nM), with increasing amounts of PARP-1. Reaction was carried out in 20 mM Tris–HCl pH 8, 30 mM KCl, 1.5 mM MgCl_2_, 1 mM DTT, 8% glycerol, 1% Phosphatase Inhibitor Cocktail I (Sigma, Milan, Italy), 5 mM NaF, 1 mM Na_3_VO_4_, 2.5 ng/mL poly [dI-dC], 50 mM ZnAc for 30 min at RT. 50 nM Cy5.5-labelled G4 was incubated with 0, 0.11, 0.22, 0.43, 0.86, and 1.72 μM PARP-1; (**C**) Plots reporting the fraction of G4 bound to PARP-1 as a function of the PARP-1 concentration. The data have been best-fitted to the Hill equation. Error bars are from two experiments; (**D**) Typical fluorescence titration of PARP-1 with G4 32R. The titration was carried out in a 500 μL quartz cuvette containing a solution of PARP-1 (60 nM) in 50 mM Na-cacodylate, pH 7.4, 100 mM KCl, 50 μM ZnAc, 1 mM DTT. G4 32R was added to the PARP-1 solution and the readings were performed 5 min after incubation. The fluorescence spectra were recorded at 20 °C between 310 and 500 nm, excitation at 295 nm. Titrations were carried out also with G4 mid1 and G4 mid2; (**E**) Plots reporting the % Trp quenching induced by increasing amounts of G4 32R, G4 mid1, and G4 mid2. Error bars are from two experiments; (**F**) ChIP showing the recruitment of PARP-1 to the G4 promoter region compared to non-G4 control ctr1 and ctr2 regions, following H_2_O_2_ treatment of Panc-1 cells. Error bars are from three experiments, (*) = *p* < 0.05.

In the past years, evidence that G4 32R is involved in transcription regulation [9,10] has been provided. G4 32R is located few helical turns upstream of TSS and is recognized by several transcription factors including PARP-1, Ku70, MAZ, and hnRNP A1 [11]. PARP-1 plays two important roles on transcription: It promotes the decondensation of chromatin [42] and it acts as a component of the transcription pre-initiation complex [43]. Due to its critical role in transcription, we focused here on PARP-1. By electrophoresis mobility-shift assays (EMSA), we explored the capacity of recombinant PARP-1 to bind to the G4s formed by G4 32R and G4 mid1, G4 mid2 (the entire G4 mid sequence showed little binding, not shown) (Figure 3A). The results obtained by incubating the oligonucleotides 5′-labelled with Cy5.5 (50 nM), mimicking the *KRAS* G4s, with increasing amounts of PARP-1 (protein:G4 ratios = 0, 2.2, 4.4, 8.6, 17.2, 34.4), are reported in Figure 3B. The interaction between PARP-1 and the G4s is rather strong, as a 4.4-fold excess of PARP-1 over G4 is sufficient to show a retarded band in the gel. In contrast, the three unstructured oligonucleotides (at PARP-1/ODN of 34.4) did not show any affinity for PARP-1 (negative control). It can be seen that protein:G4 ratios < 4 favor the formation of a 1:1 complex, whereas protein:G4 ratios > 4 favor the formation of a 2:1 complex (2 PARP-1 per G4). We reported in a plot for each G4, the fraction of bound G4 as a function the PARP-1 concentration (Figure 3C). As the curves showed a sigmoidal shape, they have been best-fitted to the Hill equation. We obtained the following *K*_D_’s values: 0.2 ± 0.03 μM for G4 32R; 0.36 ± 0.02 μM for G4 mid1; 0.37 ± 0.02 μM for G4 mid2. The three best-fits gave a cooperativity Hill coefficient of n ~ 3. In a previous study, the *K*_D_’s between PARP-1 and c-kit-1 or *Htelo* G4s, determined by surface plasmon resonance (SPR), were found to be 65 nM for c-kit-1 and 5.8 μM for *Htelo* [44]. 

As PARP-1 contains multiple Trp residues in its DNA-binding domain [44], we followed the binding of PARP-1 to G4 DNA by measuring the quenching of the Trp fluorescence upon addition of G4 to a PARP-1 solution. In Figure 3D we report a typical titration obtained by adding increasing amounts of G4 32R to a PARP-1 solution (60 nM). G4 32R quenched the fluorescence by ~50%, whereas G4 mid1 and G4 mid2 by ~30%. The percentage of quenching (measured at 345 nm) as a function of increasing amounts of G4 gave the curves shown in Figure 3E. From these plots we determined the stoichiometry of the interaction by the “tangent” method. The results pointed to a stoichiometry of two PARP-1 molecules per G4, in keeping with the results obtained with the c-kit G4 [44] and the EMSA shown in panel 3B.

Next, we explored by ChIP if PARP-1 is recruited to the G4-region of the *KRAS* promoter when the cells are treated with H_2_O_2_, i.e., after an increase of oxidative stress (Figure 3F). Panc-1 cells were treated with two doses of H_2_O_2_, 0.5 and 1.0 mM, the chromatin was extracted, sheared and used for a ChIP assay with an antibody specific for PARP-1. Input Chr (from untreated cells) and recovered Chr (from H_2_O_2_ treated cells), immuno-precipitated with antiPARP-1 Ab, were analyzed by qPCR with primers for the *KRAS* promoter G4 region and primers for the ctr1 and crt2 regions. The data obtained showed that the occupancy of the G4 region by PARP-1 compared to control ctr1 and ctr2 regions increased up to 4-fold as a function of H_2_O_2_, i.e., in a dose-response manner (Figure 3F).

We have previously reported that MAZ and hnRNP A1 too are recruited to the *KRAS* promoter G4 region rich in 8OG, in response to a H_2_O_2_ treatment [35]. Indeed, we found by a ChIP-reChiP experiment that MAZ and hnRNP A1 localize in the promoter region of *KRAS*, where the 8OG level is higher than in the control region [35]. Moreover, by EMSA we found that MAZ and hnRNP A1 bind to both G4 32R [22,23] and G4 mid sequences (Supplementary Information S7, S8).

### 2.3. G4 32R Forms A Multi-Protein Complex with PARP-1, MAZ and hnRNP A1

**Figure 4:**
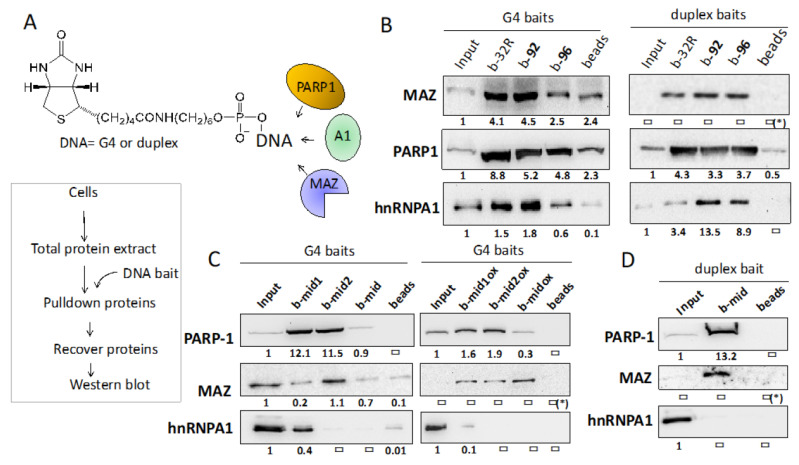
(**A**) Structure of the DNA baits used in the pull-down experiments. The scheme of the pull-down experiments is illustrated; (**B**) Pull-down with wild-type and oxidized G4 32R sequence in the G4 or duplex conformation. b-32R is wild-type G4 32R, while b-**92** bears one 8OG in a G-run, b-**96** bears two 8OG bases in the 12-nt loop (see structure in ref [35]) (Table 1). The biotinylated G4 or duplex baits (80 nM) were incubated with 80 μg of nuclear extract for 30 min at RT. The DNA bait-protein complexes formed were pulled down with streptavidin magnetic beads. The pulled-down proteins were recovered and analyzed by Western blot with anti MAZ, anti PARP-1 and anti hnRNP A1 primary antibodies and a secondary antibody conjugated to horseradish peroxidase; (**C**,**D**) Pull-down with wild-type G4 mid sequences b-mid, b-mid1 and b-mid2 or oxidized b-midox, b-mid1ox and b-mid2ox, in G4 or duplex conformation. The protocol followed is the same as in (**B**). We reported below each Western blot the band/input ratios. The uncertainty of these values from two experiments is about 20%; (*) = the band/input ratios could not be determined.

The nuclear factors recruited to the critical *KRAS* G4-region, located upstream of TSS, following a treatment with H_2_O_2_, are likely to form the transcription pre-initiation complex. To support this notion, we performed biotin-streptavidin pull-down assays with a Panc-1 nuclear extract and as DNA baits biotinylated G4 32R and G4 mid, in the wild-type or oxidized form (Figure 4B–D). The G4 baits mimicking G4 32R are wild-type b-32R and its oxidized forms b-**92** and b-**96** (Table 1). We prepared DNA baits also for G4 mid: b-mid, b-mid1 and b-mid2 and oxidized analogues b-mid^OX^, b-mid1^OX^ and b-mid2^OX^ carrying one or two 8OGs (Table 1). Each G4 bait was incubated with 80 μg of nuclear extract from Panc-1 cells. The proteins bound to the G4 bait were pulled down with streptavidin-coated magnetic beads, eluted with Laemmli buffer and analyzed by immunoblotting with antibodies specific for MAZ, hnRNP A1 and PARP-1. It can be seen that the G4 baits mimicking G4 32R (b-32R, b-**92** and b-**96**), independently from their oxidation state, pulled down all three transcription factors. This suggests that G4 32R forms a multiprotein complex, which is presumably the transcription pre-initiation complex. The eluates from the beads incubated with the nuclear extract in the absence of G4 bait, contained a small amount of proteins due to unspecific interactions between the magnetic beads coated with streptavidin and the proteins (lane “beads”). By contrast, the eluates from the beads bound to b-32R, b-**92**, and b-**96** contained a higher amount of the target proteins, due to their specific binding to the G4 structures. The nuclear factors recognize G4 32R also in the duplex conformation (obtained by hybridizing the biotinylated G4s with the complementary strand). Figure 4B shows that MAZ and PARP-1 bind to both wild-type and oxidized G4 32R in duplex conformation.

In agreement with previous studies [22], we found that whereas hnRNP A1 binds only slightly to the wild-type duplex, it binds clearly to the oxidized duplexes.

We also performed pull-down experiments with wild-type and oxidized b-mid, b-mid1 and b-mid2 as G4 baits (Table 1) (Figure 4C). These show that the 53-mer oligonucleotide b-mid, forming a putative quad-stacked G4 with a *T*_M_ = 78 °C, is not bound by the nuclear factors, whereas b-mid1 (*T*_M_ = 59 °C) and b-mid2 (67 °C) clearly bind to PARP-1. Instead, the binding of MAZ and hnRNP A1 to G4 mid1 and G4 mid2 is very weak, as the amounts of protein pulled down is comparable to those present in the input. PARP-1 binds to G4 mid1 and G4 mid2 also when they include 8OG. Some binding of MAZ to b-mid^OX^ is observed. Furthermore, and expectedly, we found that G4 mid in duplex conformation interacts with PARP-1 and MAZ but not with hnRNP A1 (Figure 4D). Together, the pull-down assays show that PARP-1, MAZ and hnRNP A1 form with the critical G4 32R sequence of *KRAS*, localized immediately upstream TSS, a transcription pre-initiation complex. G4 mid1 and G4 mid2 seems to have the function of recruiting PARP-1 in the promoter region, thus acting as a platform for the assembly of the pre-initiation complex.

### 2.4. PARP-1 Undergoes Autoparylation upon Binding to the KRAS G4 

**Figure 5:**
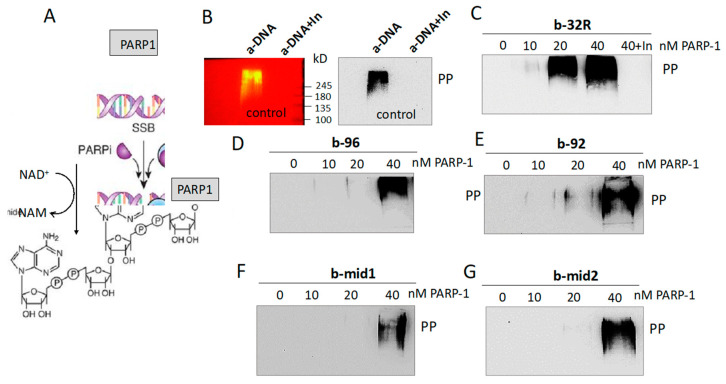
(**A**) The reaction of PARP-1 autoPARylation. NAM = nicotinamide; (**B**) AutoPARylation of recombinant PARP-1 induced by a-DNA (positive control from supplier). When Veliparib, an enzymatic inhibitor of PARP-1 (indicated with In), was added to the reaction mixture no PARylation was observed (negative control). The Western blot was performed with anti poly/mono ADP-ribose Ab and a secondary Ab conjugated to horseradish peroxidase. A membrane replica showing the protein markers (right) is also reported. PP = PARylated PARP-1; (**C**–**G**) AutoPARylation of recombinant PARP-1 (P) induced by *KRAS* G4 32R (**C**), **96** (**D**) and **92** (**E**) quadruplexes and G4 mid1 (**F**) and mid2 (**G**) quadruplexes. The mixtures contained 10, 20 and 40 nM of PARP-1 and 10 nM G4.

PARP-1 is a protein that catalyzes poly(ADP-ribosyl)ation (PARylation) of target proteins including itself (auto PARylation). Free PARP-1 consists of 6 independent domains one connected to the other by a flexible linker [45]. It has been reported that when the protein binds to DNA with a lesion through its two zinc-finger domains, it undergoes a structural change that starts the synthesis of ADP-ribose units, by using NAD^+^ (the coenzyme derived from nicotinamide, NAM) as a source of ADP-ribose. PARylation is a heterogeneous reaction in terms of length (up to 200 ADP-ribose units) and branching [46,47,48,49]. However, PARP-1 can also transfer few ADP-ribose unit or even only one [mono(ADP-ribosyl)ation] [48]. Both properties and function of PARP-1 have been summarized in a recent review [21]. In the PARylation process, DNA behaves as a positive allosteric effector, by upregulating the basal catalytic activity of PARP-1. Considering that PARP-1 associated to the *KRAS* promoter binds to G4 DNA, we wondered if the G4 structures actually behave as activators of PARP-1, as DNA with a nick or an abasic site does. There is one report in the literature addressing this issue: The autoPARylation of PARP-1 observed when the protein interacts with the ckit-1 and Htelo quadruplexes [44]. To test if the *KRAS* G4s activate recombinant PARP-1, we performed in vitro autoPARylation assays (Figure 5).

**Figure 6:**
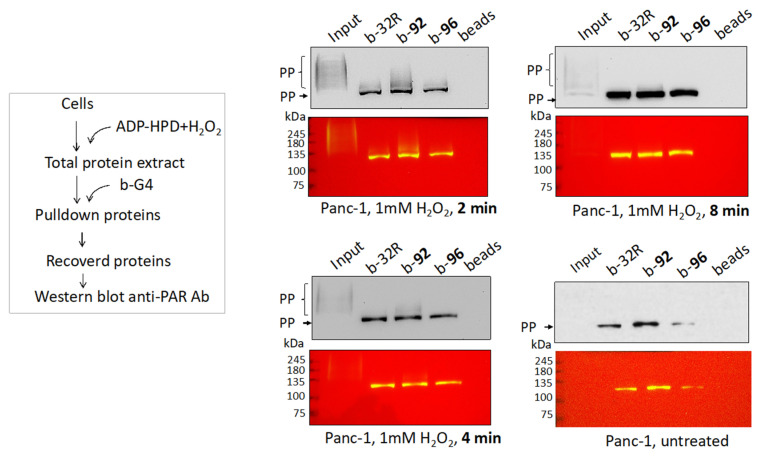
Western blots showing that b-32R and its oxidized variants b-**92** and b-**96**, pulled down PARylated proteins, from Panc-1 cells treated with H_2_O_2_. Pulled-down PARylated proteins migrate in the gel with a strong band of approximately 120–125 kDa. In contrast, the input, composed by proteins that have not been pulled down with a G4 bait, shows PARylated proteins that have molecular weights >125 kDa. Membrane replicas showing the protein markers are also reported, PP = PARylated proteins.

As positive control we used a PARP-1 activator, a-DNA (a lesioned DNA), supplied with the “PARP-1 Enzyme Activity Assay” kit (Merck Life Science, Milano, Italy). PARP-1 is a 113 kDa protein, whereas its autoPARylated form induced by a-DNA has a molecular weight > 245 kDa, suggesting an extensive auto PARylation synthesis. We then used as DNA activator *KRAS* G4s, both in the wild-type or oxidized form. The G4 activators (10 nM) were incubated with increasing amounts of PARP-1 (10, 20 and 40 nM) in a buffer containing NAD^+^. After incubation, the mixtures were blotted and analyzed with an anti poly/mono ADP-ribose Ab. The results show that the G4 activators promote to a different extent auto PARylation of PARP-1. The strongest activator is wild-type G4 32R; its oxidized variants **92** and **96** are weaker effectors. Indeed, auto PARylation induced by **92** and **96** is observed only in the presence of 40 nM PARP-1, while auto PARylation promoted by 32R is detected at 20 nM PARP-1. As expected, when Veliparib [50] (Supplementary Information S9), an inhibitor of PARP-1, is added to the reaction mixture, the auto PARylation is not observed. G4 mid1 and G4 mid2, wild-type and oxidized, promote autoPARylation of PARP-1 as well, but to a lesser extent than G4 32R. Together, these data demonstrate that both G4 32R and G4 mid1/G4 mid2, in wild-type and oxidized forms, activate PARP-1 auto PARylation. Next, we asked if PARylation is also observed within the cell context. We investigated if the proteins recruited to the *KRAS* promoter, recognizing the G4 structure of G4 32R, are PARylated. To address this issue, we followed the scheme outlined in Figure 6 Panc-1 cells were treated with H_2_O_2_ to induce guanine oxidation and stimulate the recruitment of PARP-1, MAZ and hnRNP A1 to the *KRAS* promoter. The nuclear extracts were treated with biotinylated b-32R, b-**92** and b-**96**. The pulled down samples were analyzed by Western blot with anti poly/mono ADP-ribose Ab. It can be seen that all three G4 baits pulled down PARylated proteins. Compared to untreated cells, the results show that PARylation increases with the cell exposure to H_2_O_2_. We also report in the figure the membrane replicas showing the molecular weight of the protein markers. The molecular weight of the markers loaded in the gel show that the PARylation involves a protein of ~115 kDa, suggesting that this is most likely PARP-1. Note that H_2_O_2_ gave in the input (protein extract untreated with b-G4) PARylated proteins in the range between ~130–250 kDa, while the PARylated protein captured by the G4 baits gave only a sharp band, corresponding to a protein of the size of PARP-1. This suggests that PARP-1 captured by G4 is characterized by a limited auto PARylation, if not a mono (ADP-ribosyl)ation [48].

### 2.5. The Transcription Pre-Initiation Complex Formed at G4 32R Contains Parylated PARP-1 

**Figure 7:**
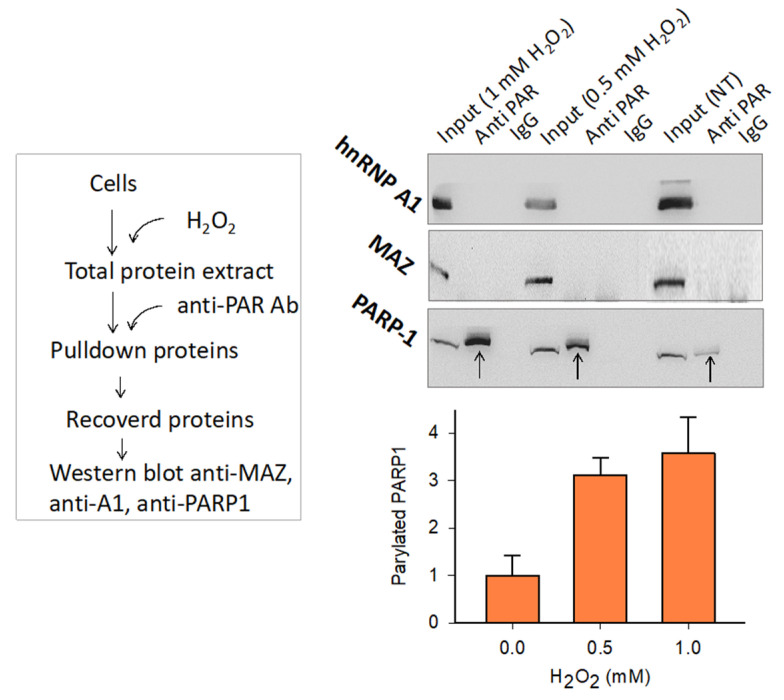
Anti-PAR immunoprecipitation assay. (Left) Experimental scheme; (Right) Western blot showing that among the PARylated proteins present in Panc-1 cells treated with 0.5 and 1 mM H_2_O_2_, there is only PARP-1 and not MAZ and hnRNP A1. The bar plot reports the ratio PARP-1 (pulled down)/PARP-1 (input) as a function of the H_2_ concentration. Error bars are from two experiments.

**Figure 8:**
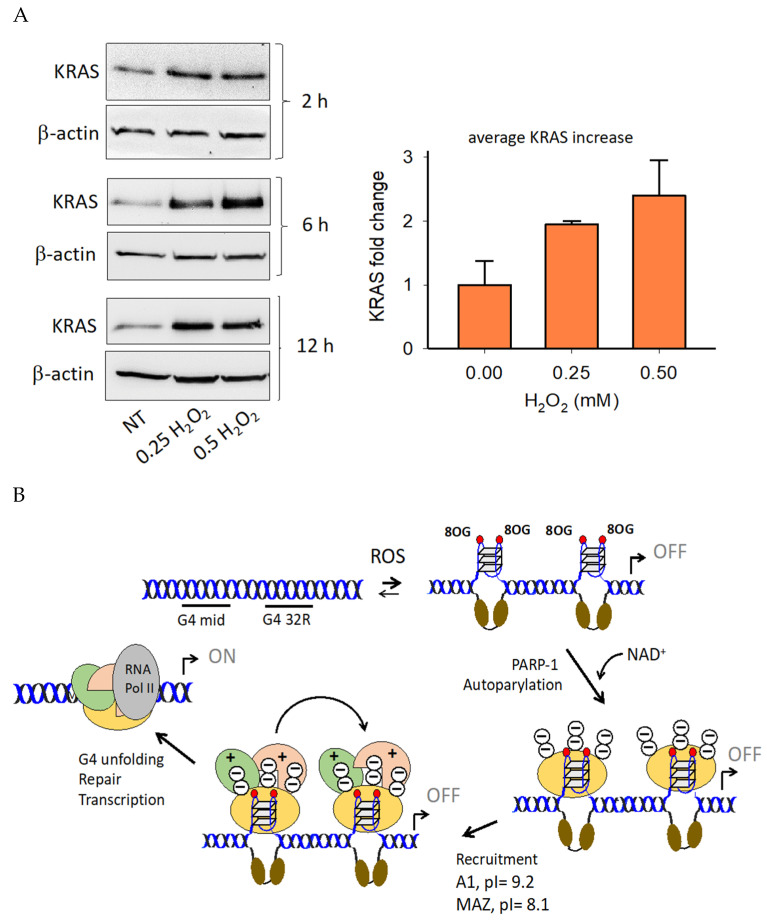
(**A**) Western blots of lysates obtained from Panc-1 cells treated with 0, 0.25, and 0.5 mM H_2_O_2_ for 2, 6, and 12 h. The cell lysates were obtained to determine the level of expressed *KRAS* protein respect to β-actin. Band density was assessed with ImageJ Software. The bar plot, reporting the average KRAS/β-actin values from the three treatments, shows that *KRAS* transcription is stimulated by ROS; (**B**) Proposed mechanism of *KRAS* activation caused by an increase of oxidative stress. A1 = hnRNP A1, pI = isolectric point.

The experiment described above showed that there are proteins bound to G4 32R that are PARylated. Although we had an indication that the main PARylated protein could be PARP-1, we wanted to unambiguously confirm it. An insight was obtained by the experiment reported in Figure 7. Nuclear extract from Panc-1 cells untreated or treated with 0.5 or 1.0 mM H_2_O_2_ was pull-down with anti poly/mono-ADP ribose Ab. The recovered PARylated proteins were separated by SDS-PAGE and blotted on nitrocellulose. Using specific antibodies we tested for the presence of PARP-1, MAZ and hnRNP A1 within the PARylated proteins. Only PARP-1 turned out to be PARylated, while MAZ and hnRNP A1 did not. The PARylation of PARP-1 occurs in a dose-response manner as, compared to untreated cells, autoPARylation increases with the amount of H_2_O_2_ used. The amount of PARylated PARP-1 in the cells treated with 0.5 and 1.0 mM H_2_O_2_ is ~4-fold more abundant than in the input (see bar plot). We concluded that upon binding to the *KRAS* G4 motif, PARP-1 undergoes autoPARylation. According to the data reported in this and previous studies, we are convinced that PARP-1 plays a key role in the mechanism controlling the expression of *KRAS* in pancreatic cancer cells. The crucial experimental observation supporting the mechanism proposed in Figure 8A is that an increase of intracellular ROS results in the upregulation of *KRAS* up to 2.5-fold, following H_2_O_2_ treatment. A similar result was obtained also when Panc-1 cells were phototreated with TMPyP4: A cationic porphyrin producing ROS upon irradiation [34]. As stated before, an increase of ROS enhances the level of 8OG in the G4-region of *KRAS* and stimulates the recruitment of PARP-1, MAZ, and hnRNP A1 to the promoter [35]. Pull-down experiments of chromatin with biotinylated G4 ligand b-6438 followed by a ChIP with anti 8OG Ab showed that 8OG and G4 DNA co-localize in the same promoter region (at ~0.2 kb resolution). If we assume that 8OG is present in the G4 motifs, because of their high guanine content, the recruitment stimulated by H_2_O_2_ increases the occupancy of the promoter G4 motifs by the transcription factors. This is indeed in keeping with pull-down and Western blot assays showing that the G4 motifs of the *KRAS* promoter form together with PARP-1, MAZ and hnRNP A1 the transcription pre-initiation complex. It is possible that 8OG behaves as an epigenetic mark for the recruitment to the promoter of the transcription factors [51,52,53]. We observed that PARP-1 binds to both G4 32R and G4 mid, either in a wild-type or oxidized form. When PARP-1 binds to G4 32R and G4 mid, it undergoes a structural change that makes the protein catalytically active, capable of auto PARylation. The autoPARylation process promoted by G4 has been confirmed in vitro, while cell-based experiments with anti poly/mono-ADP ribose and anti PARP-1 Abs showed that only few ADP-ribose units are present on PARylated PARP-1. The units of ADP-ribose attached on the surface of the protein assign a negative charge to PARP-1, which becomes anionic in nature. By contrast, MAZ and hnRNP A1, having isoelectric points (pI) of 8.1 and 9.2, respectively, are cationic in nature under physiological conditions. Thus, a possible function of PARP-1 bound to the G4 motifs is to recruit, through electrostatic interactions, the two transcription factors (Figure 8B). In this way MAZ and hnRNP A1 spatially accumulate in the promoter region where the transcription pre-initiation complex will be formed. According to this model, *KRAS* transcription is expected to be inhibited if MAZ or hnRNP A1 are suppressed. This has indeed been reported in previous works [22,24,35]. Moreover, given the important role attributed to PARP-1, the suppression or inhibition of its catalytic activity should strongly inhibit *KRAS* expression. This central issue will be addressed in the next and last section.

### 2.6. PARP-1 and G4 Are Essential for KRAS Expression 

**Figure 9:**
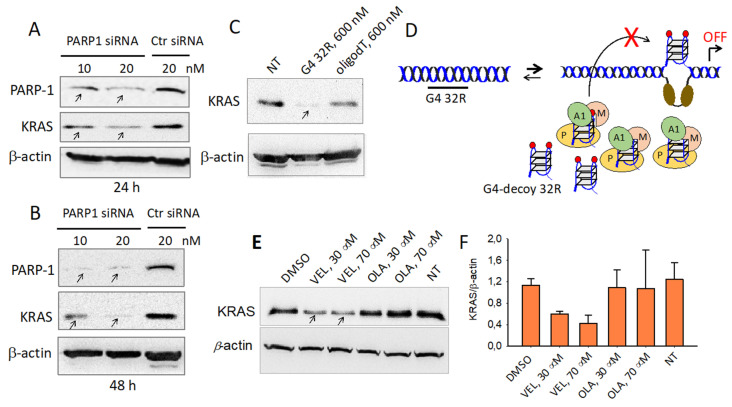
(**A**,**B**) Western blot assays showing that 10 and 20 nM PARP1 siRNA suppress PARP-1 and *KRAS* as well, in Panc-1 cells, control siRNA does not. The analysis was carried out 24 and 48 h after siRNA treatment; (**C**) Western blot showing that Panc-1 cells treated with G4 32R inhibits the expression of *KRAS* by acting as a G4 decoy which sequester the transcription factors. As a control an oligo dT was used; (**D**) Mechanism of action of G4 32R acting as a G4 decoy; (**E**,**F**) Western blot experiment. Panc-1 cells treated with 30 and 70 μM Veliparib (VEL) and Olaparib (OLA), small-molecules inhibiting the catalytic activity of PARP-1, result in >50% down-regulation of *KRAS* protein compared to control (untreated cells). Bands density were assessed with ImageJ Software. The bar plot reports KRAS/β-actin ratios. Error bars are from two independent experiments.

Besides regulating transcription through the modification of chromatin structure [17], PARP-1 interacts with the promoter of *KRAS,* thus acting as a classical transcription factor. Previous studies have reported that PARP-1 is associated with RNA Pol II in open chromatin [54]. We therefore asked what the impact of PARP-1 is on the *KRAS* promoter. We treated Panc-1 cells for 24 and 48 h with a PARP-1 specific siRNA and analyzed by Western blot the expression of PARP-1, *KRAS* and β-actin. After 48 h the siRNAs suppressed PARP-1 to ~30% of the control (siRNA ctr) and indirectly *KRAS* to ~10% of the control (Figure 9B). Such a result clearly shows that the expression of *KRAS* dramatically depends on PARP-1. We also asked if G4 DNA might play a central role in the formation of the transcription pre-initiation complex. We therefore transfected Panc-1 cells with a decoy oligonucleotide mimicking the G4 formed by G4 32R and measured by Western blot the level of *KRAS* protein (Figure 9C). The Western blot shows that 72 h after transfection with 600 nM G4 32R, the expression of the *KRAS* protein is completely suppressed. This result suggests that the exogenous G4 32R oligonucleotide transfected in the cells sequesters the transcription factors that normally form the transcription pre-initiation complex at the promoter G4 32R sequence. Finally, according to our model, transcription activation involves autoPARylation of PARP-1 bound to G4 32R and G4 mid1/G4 mid2 in order to favor the electrostatic recruitment of cationic MAZ and hnRNP A1 on the promoter. To test if PARP-1 PARylation is an important step in the mechanism activating *KRAS* transcription, we used inhibitors of the poly/mono(ADP-ribosyl)ation catalyzed by PARP-1, Olaparib and Veliparib [50,55]. Panc-1 cells were treated with 30 and 70 μM Olaparib and Veliparib for 48 h and the levels of the *KRAS* and β-actin proteins were measured by Western blot. The results reported in Figure 9E,F indicate that Veliparib decreases the expression of *KRAS* by ~60% compared to the control (untreated cells). This behavior is fully consistent with our hypothesis that the autoPARylation of PARP-1 plays a critical role in activating the expression of the *KRAS* oncogene under oxidative stress. 

## 3. Materials and Methods

### 3.1. Oligonucleotides

The non-modified oligonucleotides used in this study have been purchased from Microsynth-AG, Balgach, Switzerland. 8-oxoG-substituted oligonucleotides were synthesized from 8-oxo-dG CEP from Berry & Associates in 1-μmol scale on solid support by standard procedure, except using concentrated ammonia in the presence of 2-mercaptoethanol (0.25 M) in the deprotection step, as described by Bodepudi et al. [56]. The oligonucleotides were purified by reverse-phase high pressure (or high performance) liquid chromatography on a Water system 600, equipped with a C18 column (XBridge OST C18, 19 × 1000 mm, 5 μm). The composition of the oligonucleotides was verified by Matrix Assisted Laser Desorption Ionization-Time of Flight (MALDI-TOF). Sequences are reported in Table 1.

### 3.2. Cell Culture

Human pancreatic ductal adenocarcinoma cells (Panc-1), bearing a heterozygous missense G12D mutation in the *KRAS* gene, were maintained in exponential growth in Dulbecco’s modified Eagle’s medium High Glucose (DMEM-High Glucose) containing 100 U/mL penicillin, 100 mg/mL streptomycin, 20 mM L-glutamine and 10% fetal bovine serum. All reagents were purchased from EuroClone S.p.A., Pero (Milano), Italy.

### 3.3. Chromatin Immunoprecipitation and Quantitative PCR

ChIP was carried out by using the ChIP-IT^®^ Express Shearing Kit (Active Motif, Carlsbad, CA, USA). In brief, 8 × 10^5^ Panc-1 cells were seeded in 6-well plates and after 24 h some plates were treated with 1 or 0.5 mM H_2_O_2_ for 30 min in serum-free DMEM-High Glucose. Then, the cells were washed with phosphate buffered saline (PBS) and fixed for 10 min in serum-free DMEM-High Glucose containing 1% formaldehyde. After fixing, the cells were washed with cold PBS and Glycine Stop-Fix Solution was added to arrest the fixing reaction. The cells were then washed again with cold PBS, treated with Scraping Solution and centrifuged at 720 g for 10 min at 4 °C. The pellet was re-suspended in ice-cold lysis buffer supplemented with PMSF and PIC (protease inhibitor cocktail) and incubated for 30 min on ice. The cells were transferred to an ice-cold dounce homogenizer for 10 strokes to release the nuclei. The homogenate was centrifuged for 10 min at 2400 g at 4 °C, to pellet the nuclei. The nuclei were re-suspended in Shearing Buffer and the chromatin sheared by sonication (10 × (30 s pulse on/30 s pulse off)) on Bioruptor Plus (Diagenode, Seraing, Belgium) into DNA fragments of about 300–400 bp. The sheared chromatin was centrifuged at maximum speed for 15 min, 4 °C. The chromatin concentration was determined with a spectrophotometer by UV absorption at 260 nm. Ten micrograms of chromatin were treated overnight at 4 °C with 1 μg PARP-1 specific antibody (Santa Cruz Biotechnology, Heidelberg, Germany). In addition to the antibody, the mixtures were added with Protein G Magnetic Beads, ChIP buffer-1 and PIC, following the Active Motif Kit protocol. After overnight incubation at 4 °C, the mixtures were spun and the chromatin bound to the antibody collected with a magnetic bar. The collected beads were washed once with ChIP buffer-1 and twice with ChIP buffer-2. The beads were then re-suspended in Elution Buffer AM2 and let to incubate on shaking for 15 min at room temperature. The beads were treated with Reverse Crosslinking Buffer and the supernatant with the chromatin was collected. 1 μg of chromatin was used as “input” to assess G4 enrichment after incubation with Ab-PARP1; in addition to the chromatin, the mixtures were added with ChIP Buffer-2 and 10 mM NaCl. Both input and immuno-precipitated chromatins were heated at 95 °C for 15 min, added with Proteinase K and incubated at 37 °C for 1 h. Finally, Proteinase K Stop Solution was added to halt the reaction. The DNA fragments were amplified by qPCR using primers specific for genomic *KRAS* (accession number NG_007524):

G4-plus 5′-GTACGCCCGTCTGAAGAAGA-3′ (nucleotides (nt) 4889–4908, 0.2 μM),
G4-minus 5′-GAGCACACCGATGAGTTCGG-3′ (nt 4958–4977, 0.1 μM),
Ctr1-plus 5′-ACAAAAAGGTGCTGGGTGAGA-3′ (nt 12–32, 0.2 μM),
Ctr1-minus 5′-TCCCCTTCCCGGAGACTTAAT-3′ (nt 248–268, 0.2 μM),
Ctr2-plus 5′-CTCCGACTCTCAGGCTCAAG-3′ (nt 7536–7555, 0.15 μM),
Ctr2-minus 5′-CAGCACTTTGGGAGGCTTAG-3′ (nt 7692–7711, 0.15 μM).

The *KRAS* G4 region is located upstream TSS in the non-coding strand, ctr1 is about 5 k-bp upstream TSS while ctr2 is in an intron, about 3 k-bp downstream TSS. The % GC contents of the G4, ctr1, and ctr2 regions are 65, 44 and 59%, respectively. The lower GC content of ctr1 is only apparent because it contains three 29-mer sequences with a GC content of 55, 59, and 60%. The G4, ctr1, and ctr2 sequences are within chromatin fragments of 300–400 bp; the length of the amplified sequences are 91, 256, and 176 bp, respectively. The qPCR reactions were carried out with a CFX-96 real-time PCR apparatus controlled by Optical System software (version 3.1) (Bio-Rad Laboratories, Segrate (Milano), Italy) on 1 μL of immuno-precipitated chromatin or input, which were mixed to Sybr Green mix following manufacturer instructions (Kapa Sybr Fast QPCR Mix, Kapa Biosystems, MA, USA) and primers. For G4-region amplification cycles were: 3 min at 95 °C, 40 cycles 30 s at 95 °C and 40 s at 59 °C. For controls amplification cycles were: 3 min at 95 °C, 40 cycles 10 s at 95 °C and 30 s at 57 °C (ctr1) or 61 °C (ctr2). All reactions have been validated before amplification for each target and couple of primers. The Ct-values (number of cycles required for the fluorescent signal to cross the threshold) given by the instrument (Bio-Rad, CFX96 Real-time PCR detection System) were used to evaluate the difference between sample and input. The adjusted Ct input was obtained by Ct input – log2 (input dilution factor). Then ΔCt = Ct sample − Adjusted Ct input. The % Input for each sample was calculated as follows: % Input = 100 × 2^−ΔCt^. The % Input was obtained for the G4 region and also for the non-G4 regions (ctr1 and ctr2). The enrichment in 8-oxoG or MAZ or hnRNP A1 or OGG1 of the G4 region respect to the non-G4 regions was determined by the ratio:

[% Input (G4 region)]/[% Input (non-G4 region)]

### 3.4. Mobility Shift Assays

Recombinant PARP-1 was purchased by Antibodies-online GmbH, Germany and provided as powder; the lyophilized protein was reconstituted with dH_2_O following manufacturer instructions.

Recombinant MAZ and hnRNP A1 proteins were obtained as reported in Supplementary Information S1. Before EMSA, 5′Cy5.5 G4 oligonucleotides (G4 32R, G4 mid1 and G4 mid2) were allowed to form their structure in 50 mM Tris–HCl, pH 7.4, 100 mM KCl, heated at 95 °C for 5 min and incubated overnight at RT. Cy5.5-oligonucleotides (50 nM) were treated for 30 min at 25 °C with different amounts of PARP-1/hnRNP A1/MAZ, ([protein]/[oligonucleotide] ratios reported in the text) in 20 mM Tris–HCl pH 8, 30 mM KCl, 1.5 mM MgCl_2_, 1 mM DTT, 8% glycerol, 1% Phosphatase Inhibitor Cocktail I (Merck Life Science, Milano, Italy), 5 mM NaF, 1 mM Na_3_VO_4_, 2.5 ng/mL poly [dI-dC], 50 μM ZnAc. After incubation, the reaction mixtures were loaded in 5% TB (1×) polyacrylamide gel and run at 300 V, 50 mA, 30 W for 2 h at 20 °C. After running, the gel was analyzed with Odyssey CLx Imaging System (Li-COR Biotechnology, Bad Homburg, Germany).

### 3.5. Nuclear Extract and Biotin-Streptavidin Pull Down Assay 

To obtain nuclear extracts, 6 plates of 15 cm diameter of Panc-1 cells at a given confluence were collected and exposed to 0.5 μM ADP-HPD, an inhibitor of PARG enzyme (Merck Life Science, Milano, Italy) for 1 h. Subsequently, the cells were washed with PBS and treated with 1mM H_2_O_2_ in serum free DMEM-High Glucose for 2, 4, and 8 min. The cells were collected in PBS buffer, centrifuged at 800 g for 10 min at 4 °C. Next, the cells were resuspended in hypotonic buffer (10 mM HEPES-KOH, pH 7.9, 1.5 mM MgCl_2_, 10 mM KCl, 0.2 mM PMSF, 0.5 mM DTT, 5 mM NaF, 1 mM Na_3_VO_4_) and kept in ice for 10 min. The swollen cells were homogenized with a Dounce homogenizer and the nuclei, pelleted by centrifugation and resuspended in low-salt buffer (20 mM HEPES-KOH, pH 7.9, 25% glycerol, 1.5 mM MgCl_2_, 20 mM KCl, 0.2 mM EDTA, 0.2 mM PMSF, 0.5 mM DTT). Release of nuclear proteins was obtained by the addition of a high-salt buffer (low-salt buffer containing 1.2 M KCl). Protein concentration was measured according to the Bradford method. Biotinylated 32R, **92**, **96**, mid1, mid2, mid, midox mid1ox and mid2ox were folded in 50 mM Tris-HCl, pH 7.4 and 100 mM KCl by heating the solutions at 95 °C for 5 min and successive incubation at RT overnight. The corresponding duplexes were obtained by annealing (5 min at 95 °C and overnight at RT) the G4 oligonucleotides with the complementary strands in 50 mM Tris–HCl pH 7.4, 100 mM NaCl. 80 μg of Panc-1 nuclear extract were incubated for 30 min at RT with 80 nM biotinylated 32R, **92**, **96**, mid1, mid2, mid, midox, mid1ox and mid2ox in 20 mM Tris–HCl, pH 7.4, 150 mM KCl, 8% glycerol, 1 mM DTT, 0.1 mM ZnAc, 5 mM NaF, 1 mM Na_3_VO_4_ and 2.5 ng/μL poly[dI-dC]. Then 80 μg of Streptavidin MagneSphere Paramagnetic Particles (Promega Italia, Milano, Italy) were added and let to incubate for 30 min at RT. The beads were captured with a magnet and washed two times. The proteins were denatured and eluted with Laemmli buffer (4% SDS, 20% glycerol, 10% 2-mercaptoethanol, 0.004% bromophenol blue and 0.125 M Tris–HCl).

### 3.6. Western Blots

Protein samples were separated in 10–12% SDS-PAGE and blotted onto nitrocellulose membrane at 70 V for 2 h. The nitrocellulose membrane was blocked for 1 h with 5% fat dried milk in PBS and 0.1% Tween (Merck Life Science, Milano, Italy) at room temperature. The primary antibodies used were: Anti-MAZ (clone 133.7, IgG mouse, Santa Cruz Biotechnology, Dallas, TX, USA), anti-hnRNP A1 (clone 9H10, IgG mouse, Merck Life Science, Milano, Italy), anti-PARP-1 (polyclonal antibody, IgG rabbit, Cell Signalling Technology, Leiden, The Netherlands), anti-PAR (Poly/Mono-ADP Ribose (E6F6A) Rabbit mAb #83732, Cell Signalling Technology, Leiden, The Netherlands), anti-β-actin [Anti-Actin (Ab-1) Mouse mAb (JLA20)], (Merck Life Science, Milano, Italy), anti-*KRAS* (Mouse monoclonal Anti-KRAS, clone 3B10 2F2, Merck Life Science, Milano, Italy). The membranes were incubated overnight at 4 °C with the primary antibodies, then washed with 0.1% Tween in PBS and incubated for 1 h with the secondary antibodies conjugated to horseradish peroxidase: Anti-mouse IgG (diluted 1:5000), anti-rabbit IgG (diluted 1:5000) and anti-mouse IgM (diluted 1:5000) (Merck Life Science, Milano, Italy). The signal was developed with Super Signal^®^ West PICO, and FEMTO (Thermo Fisher Scientific, Waltham, MA, USA) and detected with ChemiDOC XRS, Quantity One 4.6.5 software (Bio-Rad Laboratories, Segrate, (Milano), Italy). Bands quantification was performed with the ImageJ software.

### 3.7. PAR Immunoprecipitation Assay 

Panc-1 cells were seeded onto 15 cm diameter plates. At a confluence of 80%, the cells were treated with 1 and 0.5 mM H_2_O_2_ in serum-free DMEM High Glucose medium for 30 min. Then the nuclear proteins were extracted and quantified as described in the “Nuclear extract and biotin-streptavidin pull down assay”. For immunoprecipitation, 1.5 mg of Protein A-Dynabeads (ThermoFisher Scientific-Invitrogen, Waltham, MA, USA) were incubated with 3 μg of PAR antibody (Poly/Mono-ADP Ribose (E6F6A) Rabbit mAb #83732, Cell Signalling Technology, Leiden, The Netherlands) and 3 μg of IgG Rabbit (ThermoFisher Scientific-Invitrogen, Waltham, MA, USA) as negative control in 20 mM Tris-HCl, pH 7.4, 150 mM KCl, 8% glycerol, 1mM DTT and 0.1 mM ZnAc for 15 min at RT. After one wash with the same buffer, 80 μg of nuclear extracts were allowed to react with anti-PAR- and IgG rabbit-derivatized Dynabeads for 30 min at RT. The beads were captured with a magnet and washed twice with the same buffer. The proteins were denatured and eluted with Laemmli buffer (4% SDS, 20% glycerol, 10% 2-mercaptoethanol, 0.004% bromophenol blue and 0.125 M Tris–HCl).

### 3.8. PARP-1 AutoPARylation and Inhibition Assays 

Oligonucleotides 32R, **92**, **96**, mid1, mid2, and mid sequences were allowed to fold into G-quadruplex as reported previously. 0, 10, 20, and 40 nM rPARP-1 (Antibodies-online, Aachen, Germany) was incubated with 10 nM biotinylated sequences in 50 mM Tris-HCl, pH 7,4, 50 mM KCl, 2 mM MgCl_2_, 1 mM DTT, 50 μM ZnAc and 150 μM NAD^+^ in 15 μL final volume for each reaction. Samples include both positive (PARP-1 incubated with activating-DNA from “PARP-1 Enzyme Activity Assay”, Merck Life Science, Milano, Italy) and negative control (PARP-1 incubated with 400 μM Veliparib inhibitor, purchased from Selleckchem, Munich, Germany). Incubation was carried out for 30 min at RT and stopped with the addition of Laemmli buffer (4% SDS, 20% glycerol, 10% 2-mercaptoethanol, 0.004% bromophenol blue and 0.125 M Tris–HCl).

### 3.9. Inhibition of KRAS by G4 Decoys, siRNA and Veliparib

To evaluate *KRAS* expression by Western blot, 5 × 10^5^ cells/well were seeded onto a 6-well plate and transfected with 600 nM 32R or 600 nM oligodT (control) by jetPEI^TM^. The cells were collected 72 h after transfection in Laemmli Buffer (4% SDS, 20% glycerol, 0.125 M Tris–HCl, pH 7.4) and lysed by Bioruptor Plus (Diagenode, Seraing, Belgium) (30 s on/30 s off) repeated 20 times. The proteins in the lysates were determined by Markwell assay [57].

To evaluate the expression level of *KRAS* following siRNA or PARP-1 inhibitor treatments, we seeded Panc-1 cells (4 × 10^5^ cells/well) onto 6-well plate. After 24 h cells were exposed to either PARP-1 siRNA (Santa-Cruz Biotechnology, Heidelberg, Germany) (10 or 20 nM, 24 and 48 h) or Veliparib/Olaparib (30 or 70 μM, 48 h) (Selleckchem, Munich, Germany) diluted in DMSO. SiRNA were delivered with INTERFERin siRNA transfection reagent (Polyplus Transfection, Illkirch, France). After treatment, the cells were washed with PBS and collected in Laemmli Buffer (4% SDS, 20% glycerol, 0.125 M Tris–HCl). Cell lysates were obtained as described above. 

### 3.10. Panc-1 Nuclear Extract

Untreated or treated Panc-1 cells (8 × 10^5^) (treated with the various molecules used in this study) were seeded onto a 6-well plate. The following day, the cells were treated with 0, 0.25, and 0.5 mM H_2_O_2_ in serum-free DMEM High Glucose for 2, 6, and 12 h. The medium was then removed, the cells washed with PBS and collected in Laemmli Buffer (4% SDS, 20% glycerol, 0.125 M Tris–HCl, pH 7.4). The cells were lysed on Bioruptor Plus (Diagenode, Seraing, Belgium), by adopting as lysis protocol, 30 s on/30 s off, repeated 20 times. The lysates were quantified with Markwell assay [57].

### 3.11. UV-Melting and CD 

UV-melting was performed by using Jasco V-750 UV-visible spectrophotometer equipped with a Peltier temperature control system (ETCS-761) (Jasco Europe, Cremella, Italy). The spectra were analyzed with Spectra Manager (Jasco Europe, Cremella, Italy). The oligonucleotides (5 μM) were annealed in 50 mM Na-cacodylate, pH 7.4 and 100 mM KCl, (5 min at 95 °C, overnight at RT). The melting curves were recorded at 295 nm in a 0.5 cm path length quartz cuvette, heating (25–95 °C) at a rate of 0.5 °C/min.

Circular Dichroism (CD) spectra were obtained on a JASCO J-600 spectropolarimeter, equipped with a thermostated cell holder, with 5 μM oligonucleotide solutions in 50 mM Na-cacodylate, pH 7.4, 100 mM KCl. The spectra were recorded in 0.5 cm quartz cuvette at 25 and 95 °C and reported as ellipticity (mdeg) versus wavelength (nm). Each spectrum was smoothed and subtracted to the baseline.

### 3.12. Fluorescence Experiments

32R, mid1 and mid2 were allowed to fold into quadruplex in 50 mM Na-cacodylate, pH 7.4, 100 mM KCl, (5 min at 95 °C, overnight at RT). The Trp fluorescence was titrated by adding to a 60 nM of recombinant PARP-1 solution (500 μL) in 50 mM Na-cacodylate, pH 7.4, 100 mM KCl, 50 μM ZnAc, 1 mM DTT, increasing amounts of G4 (see plots). A solution (without PARP-1) was used as blank. The aliquots of G4 were directly added to the PARP-1 solution and reading was performed 5′ after the incubation between PARP-1 and G4s. The fluorescence was measured on a Cary Eclipse Fluorescence Spectrophotometer (Agilent Technologies Italia, Cernusco sul Naviglio, Italy), equipped with a thermostated cell holder. The spectra were recorded at 20 °C between 310 and 500 nm, excitation at 295 nm. Each spectrum was smoothed and subtracted to the baseline.

### 3.13. Biotinylated-Anthrathiophenedione Pull-Down Assay

The pull down of chromatin fragments containing G4 structures with a G4-specific ligand b-6438 is described in Supplementary Information S2.

## 4. Conclusions

In this work we have examined the role of PARP-1 in the activation of the transcription of *KRAS* in pancreatic cancer cells. The function of this oncogene is essential in PDAC, as it reprograms the glucose and glutamine metabolism with the aim of fueling an increased anabolic demand, typical of rapidly dividing cells [4,5]. Since cancer cells are characterized by a high metabolic rate, they produce higher levels of ROS than normal cells do. So, we asked what the impact of an enhanced level of ROS is in maintaining the transformed phenotype of PDAC. The starting points of our study are: (i) ROS increase the level of 8OG in the genome, in particular in the G4 motifs composed by contiguous runs of guanines; (ii) ROS stimulate the expression of *KRAS*, the oncogene to which PDAC cells are addicted [8]. Burrows and co-workers reported that the oxidation of guanine to 8OG in the VEGF G4 motif located upstream of Rluc reporter gene increases gene expression by 4-fold [51]. If this may be considered as a general behavior and if it is applicable to the *KRAS* gene too, our work describes a possible mechanism through which 8OG in *KRAS* promoter upregulates gene expression. We have hypothesized that an increase of intracellular ROS, due to endogenous (metabolism) or exogenous (oxidants) sources, may oxidize guanine into 8OG in the G4-motif of the *KRAS* promoter located upstream of TSS. The oxidized G-rich sequence provides a platform for the assembly of the transcriptional machinery [35,52,58]. We already have found that the upregulation of *KRAS* occurring in the presence of oxidative stress stimulates the expression of Nrf2 [34]: The master regulator of cellular redox which activates the detoxification program by bringing down the ROS level for optimal cell proliferation [59,60]. We present experimental evidence that the activation of *KRAS* occurs through a mechanism involving the enrichment in 8OG of the promoter region with the G4 motifs recognized by transcription factors PARP-1, MAZ, and hnRNP A1. The enrichment of 8OG in the KAS G4 32R region produces an increase in the same region of the occupancy by PARP-1, MAZ, and hnRNP A1. This is in agreement with two facts: (i) Recombinant PARP-1 binds in vitro to G4 32R and G4 mid1/G4 mid2 sequences, (ii) biotinylated G4 32R in the G4 conformation pulls down a multi-protein complex containing the three transcription factors. The binding of PARP-1 to the *KRAS* promoter activates the catalytic property of this protein, which by utilizing NAD^+^ undergoes a limited auto PARylation. This process changes the net charge of the protein, that becomes anionic. It also favours the recruitment to the promoter of cationic transcription factors necessary for *KRAS* transcription, like MAZ and hnRNP A1. Several reports have indeed assigned to PARP-1 a role in the assembly of regulatory complexes at the promoter of the genes [17,61]. In keeping with our results Chowdhury and co-worker found that zinc-finger transcription factors, such as PARP-1 and MAZ, are closely associated to the G4 motifs in the promoter of the human genes [62]. In conclusion, our study shows that the increase of *KRAS* transcription observed in the presence of enhanced levels of oxidative stress is mediated by PARP-1.

## Figures and Tables

**Table 1:** DNA sequences used in this work.

Oligonucleotide	5′ → 3′	8OG
G4 32R	AGGGCGGTGTGGGAAGAGGGAAGAGGGGGAGG	
**92**	AGGGC**G**GTGTGGGAAGAGGGAAGAGGGGGAGG ^(1) (3)^	1
**96**	AGGGCGGTGTGGGAA**G**AG**G**GAAGAGGGGGAGG ^(1) (3)^	2
G4 mid	CGGGGAGAAGGAGGGGGCCGGGCCGGGCCGGCGGGGGAGGAGCGGGGGCCGGGC	
G4 mid1	CGGGGAGAAGGAGGGGGCCGGGCCGGGC	
G4 mid2	CGGGCCGGCGGGGGAGGAGCGGGGGCCGGGC	
b-32R	b-TTTTAGGGCGGTGTGGGAAGAGGGAAGAGGGGGAGG ^(2)^	
b-**92**	b-TTTTAGGGC**G**GTGTGGGAAGAGGGAAGAGGGGGAGG ^(1) (2) (3)^	1
b-**96**	b-TTTTAGGGCGGTGTGGGAA**G**AG**G**GAAGAGGGGGAGG ^(1) (2) (3)^	2
b-mid	b-TTTTCGGGGAGAAGGAGGGGGCCGGGCCGGGCCGGCG--GGGGAGGAGCGGGGGCCGGGC ^(2)^	
b-mid1	b-TTTTCGGGGAGAAGGAGGGGGCCGGGCCGGGC ^(2)^	
b-mid2	b-TTTTCGGGCCGGCGGGGGAGGAGCGGGGGCCGGG ^(2)^	
b-mid^OX^	b-TTTTCGGGGAGAAGGAGGGGGCC**G**GGCC**G**GGCCGGCG- -GGGGAGGAGCGGGGGCCGGGC ^(1) (2)^	2
b-mid1^OX^	b-TTTTCGGGGAGAA**G**GAGGGGGCCGGGCCGGGC ^(1) (2)^	1
b-mid2^OX^	b-TTTTCGGGCC**G**GCGGGGGA**G**GAGCGGGGGCCGGGC ^(1) (2)^	2
Cy5.5-32R	Cy5.5-AGGGCGGTGTGGGAAGAGGGAAGAGGGGGAGG	
Cy5.5-mid1	Cy5.5-CGGGGAGAAGGAGGGGGCCGGGCCGGGC	
Cy5.5-mid2	Cy5.5-CGGGCCGGCGGGGGAGGAGCGGGGGCCGGGC	
Cy5.5-mid	Cy5.5-CGGGGAGAAGGAGGGGGCCGGGCCGGGCCGGCGGGGGAGGAGCGGGGGCCGGGC	
32Y	CCTCCCCCTCTTCCCTCTTCCCACACCGCCCT	
G4 mid Y	GCCCGGCCCCCGCTCCTCCCCCGCCGGCCCGGCCCGGCCCCCTCCTTCTCCCCG	
Oligo dT	TTTTTTTTTTTTTTTT	
ODN1	Cy5.5-CATCAGAAGGCTAGCAATCA	
ODN2	Cy5.5-AATAGTAATTGCTTAGCCTG	
ODN3	Cy5.5-CCTAATGCTGCTAAACTCCC	

^(1)^ Underlined G = 7,8-dihydro-8-oxoguanine (8OG); ^(2)^ b = biotin; ^(3)^ **92** and **96** = G4 32R analogues containing 8OG.

## Supplementary Materials

Supplementary Materials can be found at https://www.mdpi.com/1422-0067/21/17/6237/s1. Supplementary S1: Recombinant proteins MAZ and hnRNP A1. Supplementary S2: Pull-down assay with b-6438. Supplementary S3: Structure of the G4 formed by sequence 32R (G4-near). Supplementary S4: Putative structures formed G4 mid1 and G4 mid2. Supplementary S5: Melting curves of G4 mid, G4 mid^ox^, G4 mid1, G4 mid1^ox^, G4 mid2, G4 mid2^ox^. Supplementary S6: Structure of a G-tetrad with one G replaced by 8OG. Supplementary S7: EMSA showing the binding of hnRNP A1 to the G4 mid sequences. Supplementary S8: EMSA showing the binding of MAZ to the G4 mid sequences. Supplementary S9: Structures of PARP-1 inhibitors Veliparib and Olaparib.

## Abbreviations

*KRAS*	*Kirsten ras* gene
MAZ	MYC associated zinc finger protein
hnRNP A1	Heterogeneous nuclear ribonucleoprotein A1
PARP-1	Poly[ADP-ribose] polymerase 1
PDAC	Pancreatic ductal adenocarcinoma
8OG	8-Oxoguanine
TSS	Transcription start site
ChIP	Chromatin immunoprecipitation
PARylation	Poly(ADP-ribosyl)ation
