# Supplementary material for: Role of Poly [ADP-ribose] Polymerase 1 in Activating the Kirsten ras (KRAS) Gene in Response to Oxidative Stress
Source: Int J Mol Sci. 2020 Aug 28;21(17):6237. doi: 10.3390/ijms21176237 (PMC7504130; doi:10.3390/ijms21176237)
Supplement: Supplementary file 1 [file ijms-21-06237-s001.pdf]

Supplementary Materials

Role of poly [ADP-ribose] polymerase 1 in activating the *Kirsten ras* (*KRAS*) gene in response to oxidative stress

Giorgio Cinque[1], Annalisa Ferino[1], Erik B. Pedersen[2] and Luigi E. Xodo*[,1]

Supplementary S1: Recombinant proteins MAZ and hnRNPA1

Supplementary S2: Pull-down assay with b-6438;

Supplementary S3: Structure of the G4 formed by sequence 32R (G4-near);

Supplementary S4: Putative structures formed G4 mid1 and G4 mid2;

Supplementary S5: Melting curves of G4 mid, G4 mid^{ox}, G4 mid1, G4 mid1^{ox} , G4 mid2, G4-mid2^{ox}

Supplementary S6: Structure of a G-tetrad with one G replaced by 8OG;

Supplementary S7: EMSA showing the binding of hnRNP A1 to the G4 mid sequences;

Supplementary S8: EMSA showing the binding of MAZ to the G4 mid sequences;

Supplementary S9: Structures of PARP-1 inhibitors Veliparib and Olaparib

Supplementary Information S1: Recombinant proteins

Recombinant MAZ and hnRNP A1 proteins tagged to glutathione S-tranferase (GST) were expressed in Escherichia coli DE3/BL21 by using, respectively, plasmid pGEX-hMAZ and pGEX-hnRNPA1. The bacteria were grown at 37 °C in LB broth plus ampicillin 100 µg/mL to an A_{600} of 0.8–1.0 prior to induction with either 1.0 mM (for MAZ) or 0.1 mM (for hnRNP A1) isopropyl 1-thio-β-D-galactopyranoside. Cells carrying pGEX-hMAZ were allowed to grow with 4µM ZnAc overnight at 37°C before harvesting. The cells were centrifuged at 4230 g at 4°C, the supernatant was removed and the cells washed twice with PBS. The pellet was resuspended in a solution of PBS with 1 mM phenylmethylsulfonyl fluoride (PMSF). The bacteria were lysed by sonication (cells containing pGEX-hnRNP A1 were added with 1% Triton X-100 and incubated for 30 min on a shaker at 4 °C) added with 0,1mM PMSF and 0,5mM DTT. The lysate was then centrifuged for 30 min at 4 °C at 34500 g. Glutathione Sepharose 4B resin (GE Healthcare) (50 % slurry in PBS) was added to the supernatant from the previous step and incubated for 1 h at 4 °C on a shaker. The mix was centrifuged for 5 min at 500 g and the pellet was washed 3 times with PBS. The protein was eluted from the resin with 50mM Tris-HCl pH 8, 10mM GSH and stored at -80 °C in the same buffer added with 0,1mM PMSF and 0,5mM DTT.

Supplementary Information S2: Biotinilated-anthrathiophenedione pull-down assay

Panc-1 cells (8 x 10^5) were seeded onto a 6-well plate and after 48 h some wells were treated with 1 mM H_2O_2 for ½ h in in serum-free DMEM High Glucose. The cells were then washed with phosphate buffered saline (PBS) and fixed for 10 min in serum-free DMEM High Glucose containing 1% formaldehyde. After fixing, the cells were washed with cold PBS and added with Glycine Stop-Fix Solution to arrest the fixing reaction. The cells were washed again with cold PBS, treated with Scraping Solution and centrifuged at 2500 rpm for 10 min at 4°C. The pellet was resuspended in ice-cold lysis buffer supplemented with PMSF, PIC (protease inhibitor cocktail) and incubated for 30 min on ice. The cells were transferred to an ice-cold dounce homogenizer for 20 strokes to release the nuclei. The homogenate was centrifuged for 10 min at 5000 rpm, 4 °C, to pellet the nuclei. The nuclei were resuspended in Shearing Buffer and the chromatin sheared by sonication [10 × (30 s pulse on/30 s pulse off)] on Bioruptor Plus (Diagenode, Seraing, Belgium) into DNA fragments of about 300–400 bp. The sheared chromatin was centrifuged at maximum speed for 15 min, 4◦C. The chromatin concentration was determined with a spectrophotometer by ultraviolet (UV) absorption (260 nm) and 20 mg were folded in 100 mM KCl, 50 mM Tris-HCl, pH 7.4 (5 min at 95 °C, overnight at RT). The folded chromatin mixed with biotinylated ligand b-6438

(0.8 mM) was incubated at 4 °C for 6h. We incubated the magnetic beads, after saturation with Salmon Sperm DNA (Invitrogen, USA), with chromatin for 30 min at RT. The supernatant was removed and the beads washed three times with 50 mM Tris-HCl, pH 7.4, 50 mM KCl. We then recovered the bound chromatin with a solution 0.8 M NaCl. The following Chromatin Immunoprecipitation experiment was carried out treating the eluted chromatin overnight at 4 °C with 1 mg antibody specific for 8-oxoG (Bioss Antibodies, Woburn, MA, USA), following the Active Motif Kit protocol previously reported in "Chromatin immunoprecipitation and quantitative PCR" section.

Supplementary Information S3: Structure of the G-quadruplex formed by sequence 32R (G4-near) located in the *KRAS* promoter, upstream of TSS. The structure has been determined by DMS-footprinting (ref. 11) and NMR (ref. 30). This 1/1/12 G4 is in equilibrium with a 1/3/11 conformer (ref. 30).

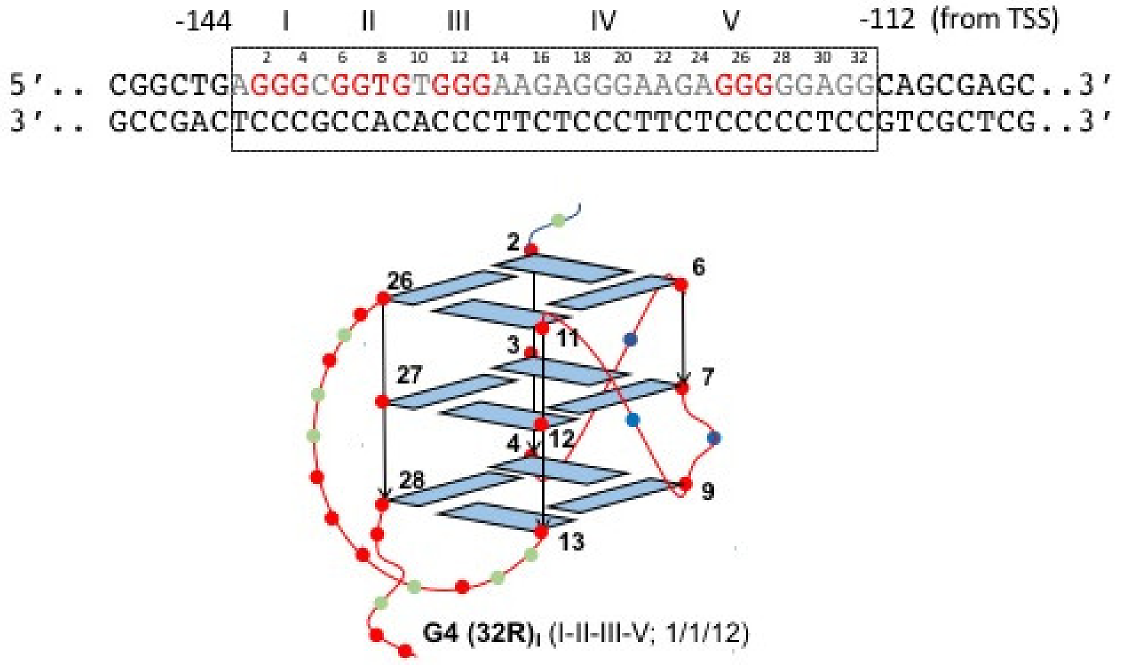

Supplementary Information S4: Putative structures of the G-quadruplexes formed by sequences G4 mid1 (T_M= 59 °C) and G4 mid2 (T_M = 67 °C) located in the *KRAS* promoter, upstream of TSS. For analogy with 32R we propose for both sequences a structure with a bulge and a 1-nt double-chain reversal loop (loops 4/1/2). This is in keeping with the fact that replacing in G4-mid1 G10 with 8OG, the T_M decreases to 56 °C, because the G-run $G^{OX}GAG$ is replaced by GGG at the 3'end

and the G4 assumes a 9/2/2 loop topology. A similar behaviour was observed with 32R (Ref. 27). As with G4 mid1, also G4 mid2 assumes a G4 with a bulge and 1-nt double-chain reversal loop (loops 2/1/6). When G7 and G15 are replaced with 8OG, the T_M goes down to 63 °C, suggesting that G7 is indeed involved in the formation of a G-tetrad.

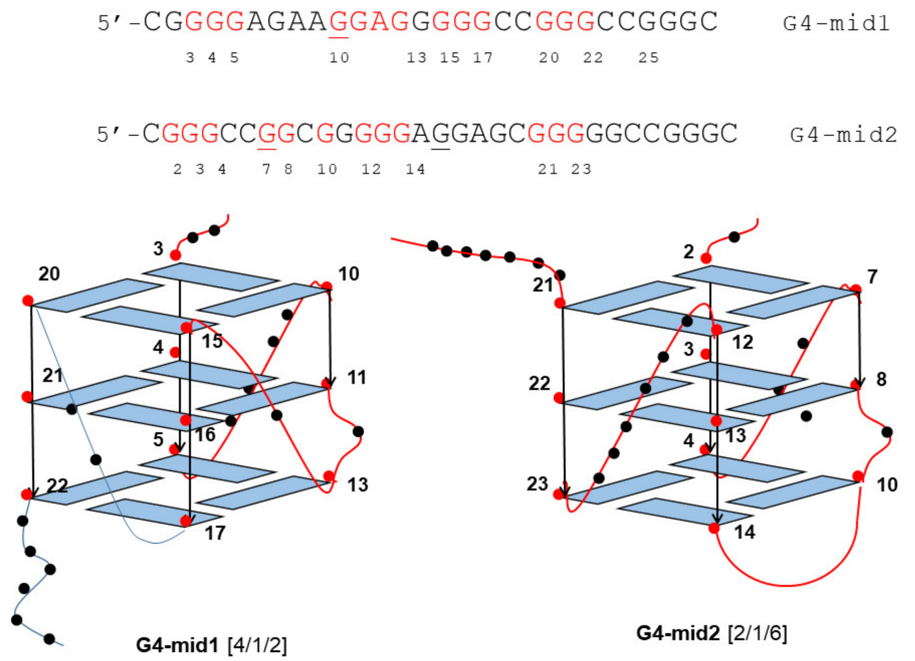

Supplementary Information S5: CD spectra and melting curves of G4 mid, G4 mid1 and G4 mid2 in 50 mM Na-cacodylate, pH 7.4 and 100 mM KCl. The T_M are: 78 °C (G4 mid), 59 (G4 mid1) and 67 (G4 mid2).

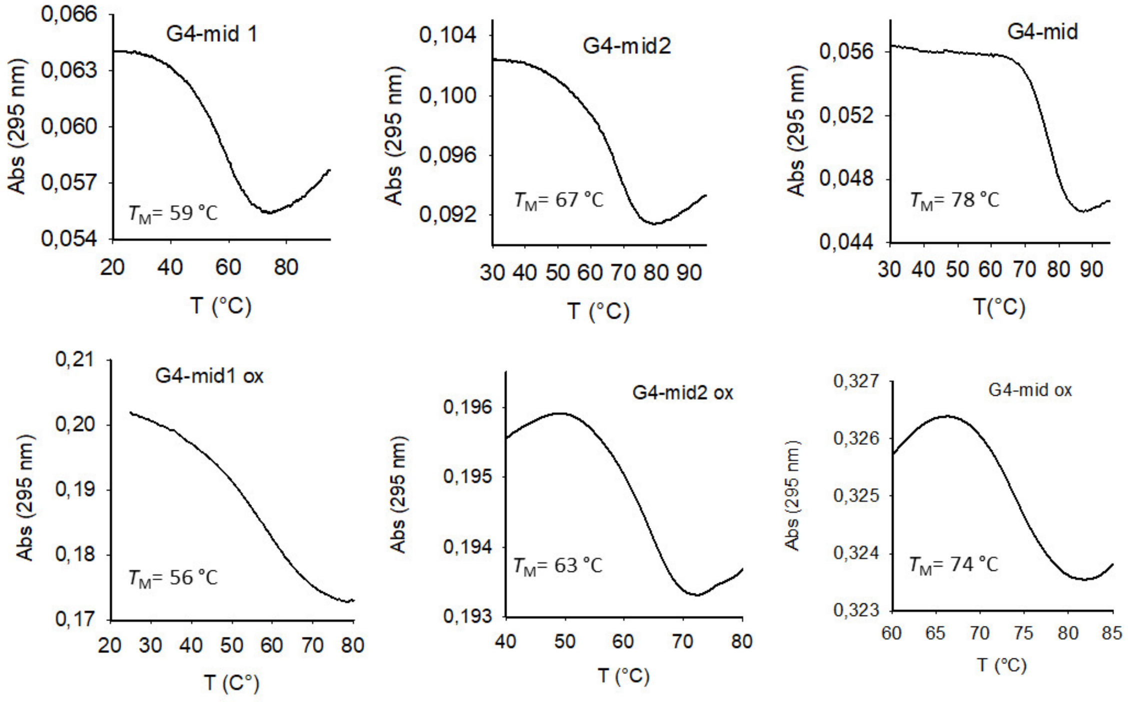

T_M of G-quadruplex structures formed by G4-near and G4-mid wild-type and oxidised (sequences are in Table 1).

32R* 62 °C	G4mid 78 °C	G4mid1 59 °C	G4mid2 67 °C
**92*** 51 °C	G4 mid^{ox} 74 °C	G4 mid1^{ox} 56 °C	G4 mid2^{ox} 63 °C
**96*** 61 °C			

*Data from ref. 27

Supplementary Information S6: 8OG destabilizes a G-tetrad: there is the loss of one Hoogsteen hydrogen bond within a G-tetrad and a potential steric hindrance between –NH2 of G and –NH- of 8OG.

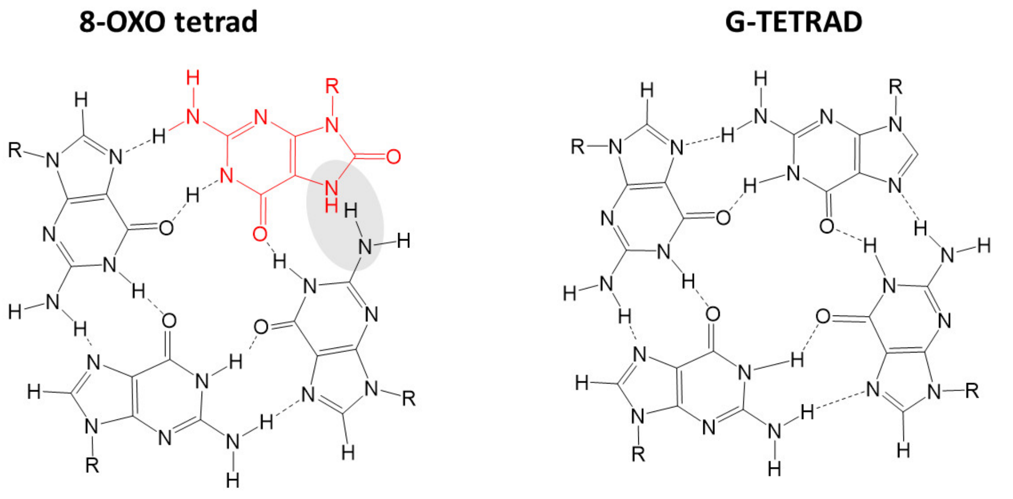

Supplementary Information S7: The results obtained incubating oligonucleotides labelled with Cy5.5 at the 5'end (50 nM), mimicking the *KRAS* G4 mid, G4 mid1 and G4 mid2, with increasing amounts of hnRNP-A1. Reaction was carried out in 20 mM Tris–HCl pH 8, 30 mM KCl, 1.5 mM $MgCl_2$, 1 mM DTT, 8% glycerol, 1% Phosphatase Inhibitor Cocktail I (Merck Life Science, Milano, Italy), 5 mM NaF, 1 mM Na_3VO_4, 2.5 ng/ml poly [dI-dC], 50 μM ZnAc for 30 min at RT. 50nM Cy5.5-labelled G4 was incubated with 0, 0.71, 1.43, 2.85 and 5.7 μM hnRNP A1.

.

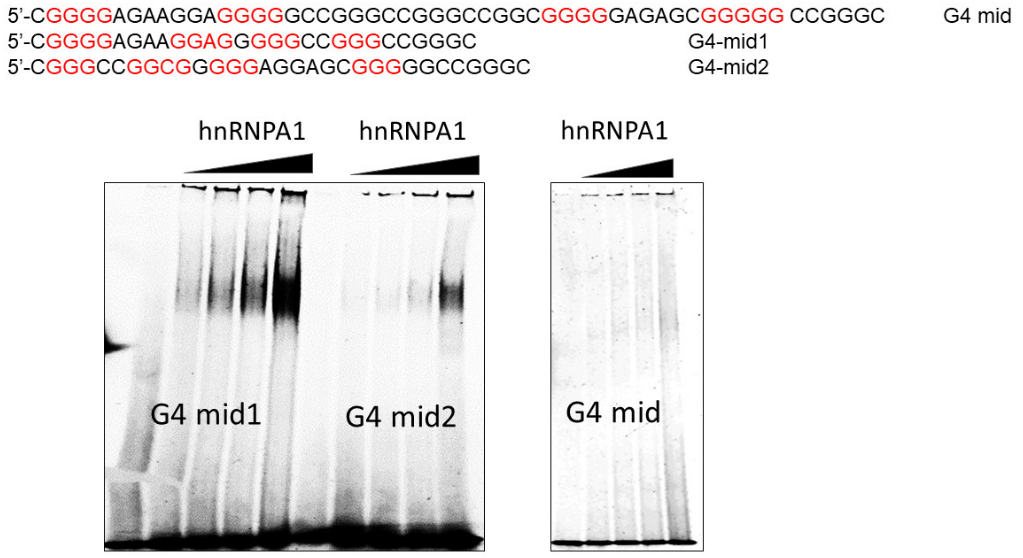

Supplementary Information S8: The results obtained incubating oligonucleotides labelled with Cy5.5 at the 5’end (50 nM), mimicking the *KRAS* G4 mid, G4 mid1 and G4 mid2, with increasing amounts of MAZ. Reaction was carried out in 20 mM Tris–HCl pH 8, 30 mM KCl, 1.5 mM $MgCl_2$, 1 mM DTT, 8% glycerol, 1% Phosphatase Inhibitor Cocktail I (Merck Life Science, Milano, Italy), 5 mM NaF, 1 mM Na_3VO_4, 2.5 ng/ml poly [dI-dC], 50 µM ZnAc for 30 min at RT. 50nM Cy5.5-labelled G4 was incubated with 0, 0.79, 1.57, 3.15 and 6.3 µM MAZ.

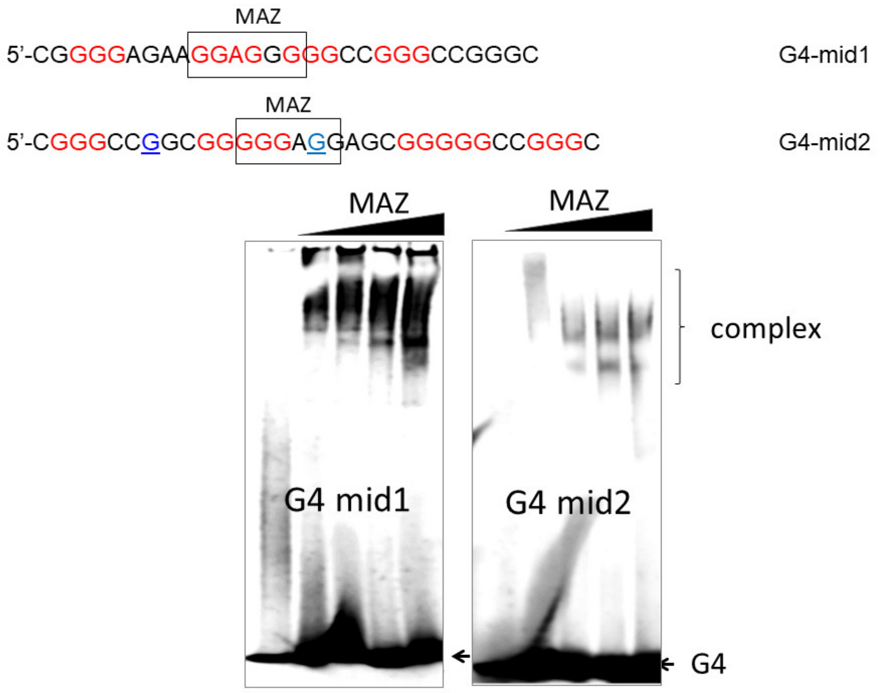

Supplementary Information S9: Structures of Veliparib and Olaparib, two PARP-1 inhibitors

VELIPARIB

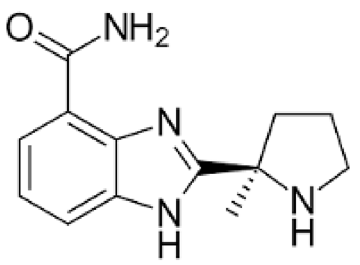

2-[(2*R*)-2-methylpyrrolidin-2-yl]-1*H*-benzimidazole-4-carboxamide

OLAPARIB

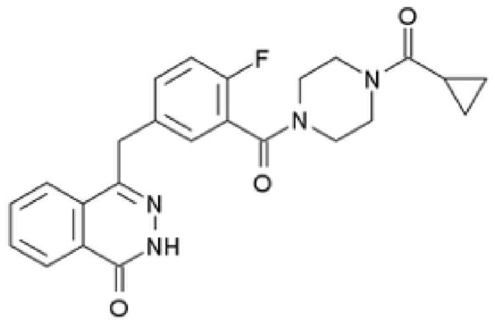

4-[(3-[(4-cyclopropylcarbonyl)piperazin-1-yl]carbonyl) –4-fluorophenyl]methyl(2H)phthalazin-1-one
